# Construction and Influence of Induced Pluripotent Stem Cells on Early Embryo Development in Black Bone Sheep

**DOI:** 10.3390/biology14050484

**Published:** 2025-04-28

**Authors:** Daqing Wang, Yiyi Liu, Lu Li, Xin Li, Xin Cheng, Zhihui Guo, Guifang Cao, Yong Zhang

**Affiliations:** 1 College of Veterinary Medicine, Inner Mongolia Agricultural University, Hohhot 010011, China; wangdaqing050789@126.com (D.W.); 15104718293@139.com (Y.L.); yunsong-5410@163.com (L.L.); 17647628761@163.com (X.L.); 17822106812@163.com (X.C.); h1454738358@163.com (Z.G.); 2 Animal Embryo and Developmental Engineering Key Laboratory of Higher Education, Institutions of Inner Mongolia Autonomous Region, Hohhot 010011, China; 3 Inner Mongolia Autonomous Region Key Laboratory of Basic Veterinary Medicine, Hohhot 010011, China; 4 College of Life Sciences, Inner Mongolia Agricultural University, Hohhot 010011, China; 5 College of Life Sciences, Inner Mongolia University, Hohhot 010021, China

**Keywords:** black bone sheep, pluripotent stem cells, piggyBac vector, cellular reprogramming

## Abstract

The piggyBac+TET-on transposon induction system has a high efficiency in integrating exogenous genes in multiple cell types, can precisely integrate to reduce genomic damage, has a flexible gene expression regulation, and a strong genetic stability. When used in conjunction with somatic cell nuclear transfer experiments, it can precisely and effectively reveal the intrinsic mechanisms of early biological development. This study successfully reprogrammed black-boned sheep fibroblasts (SFs) into induced pluripotent stem cells (iPSCs) using the piggyBac+TET-on transposon system and investigated their impact on early embryonic development. Seven exogenous reprogramming factors (bovine OCT4, SOX2, KLF4, cMyc, porcine NANOG, Lin-28, and SV40 Large T) were delivered into SFs, successfully inducing iPSCs. A growth performance analysis revealed that iPSC clones exhibited a raised or flat morphology with clear edges, positive alkaline phosphatase staining, and normal karyotypes. The transcriptome analysis indicated a significant enrichment of iPSCs in oxidative phosphorylation and cell proliferation pathways, with an up-regulated expression of the ATP5B, SDHB, Bcl-2, CDK1, and Cyclin D1 genes and a down-regulated expression of BAX (*p* < 0.05). Somatic cell nuclear transfer experiments demonstrated that the cleavage rate (85% ± 2.12) and blastocyst rate (52% ± 2.11) of the iPSCs were significantly higher than those of the SFs (*p* < 0.05). The detection of trilineage marker genes confirmed that the expression levels of endoderm (DCN, NANOS3, FOXA2, FOXD3, SOX17), mesoderm (KDR, CD34, NFH), and ectoderm (NEUROD) markers in iPSCs were significantly higher than in SFs (*p* < 0.01). The findings demonstrate that black-boned sheep iPSCs possess pluripotency and the potential to differentiate into all three germ layers, revealing the mechanisms by which reprogrammed iPSCs influence early embryonic development and providing a critical foundation for research on sheep pluripotent stem cells.

## Simple Summary

The piggyBac+TET-on transposon system was used to reprogram black bone sheep fibroblasts into iPSCs and explore the mechanism of their influence on early embryonic development. The iPSC clones have a good morphology, positive alkaline phosphatase staining, and normal karyotype. The transcriptome analysis showed that it was significantly enriched in oxidative phosphorylation and cell proliferation pathways, and the expression of related genes was significantly different from that of fibroblasts. In the somatic cell nuclear transfer experiment, the cleavage rate and blastocyst rate of the iPSC were higher than those of fibroblasts, and the difference was obvious. The expression of three-layer marker genes in iPSCs was much higher than that in fibroblasts. These results indicated that the obtained iPSCs had pluripotent and triploblastic development potential and revealed the mechanism of action of the reprogrammed iPSCs on early embryonic development, which laid a solid foundation for the study of pluripotent stem cells in sheep.

## 1. Introduction

The piggyBac+Tet-on transposon system is a powerful tool in the field of genetic engineering, integrating the PiggyBac transposon system with the Tet-on inducible gene expression system. The PiggyBac (PB) transposon, derived from Lepidoptera insects, belongs to the second class of eukaryotic transposons, with a length of 2476 bp, containing inverted terminal repeat sequences (ITRs) and open reading frames (ORFs). It transposes via a “cut–paste” mechanism, featuring a high efficiency, wide host range, precise excision and transposition, and low dependence on host factors [1]. The Tet-on system utilizes prokaryotic regulatory elements to precisely control the expression of exogenous genes in eukaryotic cells. In the absence of tetracycline or its analogs (such as doxycycline), the tTS fusion protein binds to the tetracycline response element promoter (TRE), inhibiting gene transcription. In the presence of tetracycline or its analogs, the rtTA fusion protein binds to the TRE, activating transcription [2]. The piggyBac+Tet-on transposon system consists of two vectors: an auxiliary plasmid encoding the transposase and a transposon plasmid. After the co-transfection into target cells, the transposase from the auxiliary plasmid recognizes the TRs of the transposon and inserts the region containing the TRs and the intermediate gene expression cassette into the host genome. By adding or removing tetracycline and its analogs, the expression of the target gene in the host cells can be regulated. In human pluripotent stem cells (hPSCs), this system can induce the expression or silencing of target genes at specific times, facilitating the study of the role of genes in cell differentiation and development processes. For instance, by constructing this system in hPSCs and using doxycycline to activate specific genes, the impact on cell differentiation can be explored [3].

Pluripotent stem cells (PSCs) have the capacity to differentiate into various cell types, making them invaluable for studying differentiation mechanisms and regulations. Induced pluripotent stem cells (iPSCs) exhibited both a plasticity and self-renewal ability [4], providing unique advantages for animal production. The successful establishment of pluripotent stem cell lines suggests promising new methods for animal breeding and production [5].

Takahashi et al. confirmed that four factors, OCT4, SOX2, KLF4, and cMyc (OKSM), are sufficient to reprogram somatic cells into PSCs [6]. Lei et al. proposed that viral vectors can introduce OSKM and other genes into somatic fibroblasts (SFs) at various developmental stages, leading to the expression of pluripotency markers and the formation of embryoid bodies (EBs) and teratomas [7]. Subsequent studies have introduced pluripotency genes into sheep cells using the PiggyBac transposon system and microRNAs [8]. Research involving diverse culture media and pluripotency markers has shown that most sheep iPSCs can form EBs, while some can also generate teratomas [9]. Currently, all established goat iPSCs originated from fetal, embryonic, or SF cells [10]. In most cases, the reprogramming is achieved by delivering OSKM or its combination with other pluripotency genes into cells with retroviral vectors or lentiviral vectors [11]. The primary goat iPSCs exhibited a colony morphology reassembling mice iPSCs, exhibited the positive staining of alkaline phosphatase (AP) [12], and expressed a specific pluripotency. Although most goat iPSCs are able to develop into EBs, they rarely form teratomas in vivo [13]. Directed differentiation in goat iPSCs has been shown to produce trophoblast cells, yolk sac endodermal cells, and neuronal cells [14]. Genetic studies have highlighted that key transcription factors, including OCT4, SOX2, and NANOG [15], are essential for maintaining pluripotency and promoting early embryonic development. Meanwhile, Lin-28, a reprogramming factor, can exert a collaborative function together with OCT4, SOX2, and NANOG in inducing pluripotency [16].

This study selects seven exogenous transcription factors: OCT4, SOX2, KLF4, cMyc, NANPG, Lin-28, and SV40 Large T. The factors were used to reprogram black bone sheep SFs into iPSCs through the piggyBac+TET-on transposon system. A transcriptome analysis was conducted to explore differences in metabolic pathways and compare the expression levels of key markers. Furthermore, oocyte nuclear transplantation was used to examine the cleavage rate of iPSCs, and the quantitative analysis of lineage-specific markers was carried out to evaluate their underlying developmental capability. The objective of this study is to establish iPSCs from black-boned sheep with developmental potential and to investigate their mechanisms in early embryonic development. This research aims to provide a theoretical foundation for further studies on ovine pluripotent stem cells.

## 2. Materials and Methods

### 2.1. Materials

All the black bone sheep fibroblasts used in this experiment were isolated and cultured from the cell bank of Animal Embryo Development Laboratory, Inner Mongolia Agricultural University, and frozen in a liquid nitrogen tank. STO cells are derived from the cell resource bank of Inner Mongolia Saikxing Research Institute.

The 15 mL centrifuge tube (KG2614), 150 mm cell culture dishes (430167), and T25 cell culture flask (430639) were purchased from Corning Company (Corning, New York, NY, USA). Dulbecco’s Phosphate-Buffered Saline (DPBS) (C3590-0500) and Dulbecco’s Modified Eagle Medium/Nutrient Mixture F12 media (DMEM/F12) (11320033) were purchased from Gibco (New York, NY, USA). Dimethyl Sulfoxide (DMSO) (D-2650), mitomicin C (M5353), and colchicine (855774) were purchased from Sigma-Aldrich (Burlington, New York, NY, USA).

The primers used were designed and synthesized by Sangon Biotech (Shanghai, China).

The cell culture medium formulations are as follows (Table 1, Table 2 and Table 3).

### 2.2. iPSC Preparation and Growth Performance Identification of Black Bone Sheep

#### 2.2.1. Detection of iPSC Electrotransfection Efficiency of Black Bone Sheep

Thaw the 6th generation of female black-boned sheep skin SFs for culture, take 1 × 10^6^ cells, and obtain a cell suspension. Centrifuge at 1500 rpm for 3 min, discard the supernatant. Take 100 μL of electroporation solution mixture and 2 μg of pmaxGFP™ vector, mix well, and add to the centrifuge tube to resuspend the cells. After mixing, gently transfer the mixture into the electroporation cuvette, then place the cuvette into the LONZA (New York, NY, USA) electroporation device. Set different electroporation programs to perform electroporation, and compare the efficiency of GFP fluorescence in adherent cells after 24 h.

#### 2.2.2. Induction of Induced Pluripotent Stem Cells of Black Bone Sheep

One day before electroporation, thaw STO feeder cells into a 100 mm cell culture dish, add M10 medium, and culture at 37 °C with 5% CO_2_. On the day of cell electroporation, replace the culture medium with M15 + doxycycline (to induce stem cell differentiation). Prepare the following plasmids: 2.0 μg PB–TRE-bOSKM (bovine OCT4, SOX2, KLF4, and cMYC), 1.0 μg PB–TRE–pNhL (porcine NANOG and human LIN28), 1.0 μg PB–TRE–sLargeT (simian virus Large T), 1.0 µg PB–EF1a–transposase, and 1.0 µg PB–EF1a–rTTA. Mix these with 100 μL of electroporation solution and let stand at room temperature for later use.

When the cell confluence reaches approximately 80%, collect the cell mixture and perform electroporation using the optimal electroporation program. Add 1 mL of pre-warmed M10 medium to the electroporation cuvette, then transfer the cell suspension from the cuvette into a 1.5 mL centrifuge tube using a specialized pipette. Gently mix and transfer one-quarter of the mixture into a 100 mm culture dish containing M10 medium, and place it in the cell culture incubator (37 °C, 5% CO_2_). Change the medium after 24 h, and then every other day thereafter. After 7–10 days, use a suitable glass needle to detach the cell clones from the STO feeder layer, aspirate them, and digest them with trypsin. After stopping the digestion, directly transfer the mixture into a 24-well plate pre-seeded with STO feeder cells for further culture and observation.

#### 2.2.3. Pluripotent Gene Test

When the cell clones grow to a size of 10–15 mm^2^, digest the cells and collect the cell suspension. Centrifuge the suspension in a 1.5 mL enzyme-free centrifuge tube, resuspend the cell pellet in DPBS, and centrifuge again. Discard the supernatant, label the samples, and store them at −80 °C for future use.

To extract total RNA, take out the cell samples and centrifuge them, then discard the supernatant. Add cell lysis buffer RA2, mix thoroughly by inverting until the lysis buffer becomes clear, and transfer the entire liquid to the inner sleeve. Centrifuge and discard the filtrate. Add wash buffer to the inner sleeve, centrifuge, and discard the filtrate. Perform an empty spin for 1 min. Place the inner sleeve into a new 1.5 mL RNase-free centrifuge tube, add elution buffer to the center of the membrane, and centrifuge for 1 min to obtain total RNA. Verify the OD value of the RNA, and select RNA samples with an OD260/280 ratio between 1.8 and 2.1 and a concentration of at least 40 ng/μL for subsequent experiments. Reverse transcribe the RNA into cDNA according to the instructions of the Vazyme HiScript QRT SuperMix for qPCR kit (R223-01, Wuhan, China). The 6th generation SFs of the black-bone sheep were used as the control group, and the 6th generation siPSCs as the experimental group. Validate the expression levels of endogenous and exogenous pluripotency genes and observe cell growth (Table 4 and Table 5).

#### 2.2.4. Alkaline Phosphatase Staining

The transfected cells were placed in the culture medium containing STO cells for 2 consecutive days, then rinsed with DPBS twice, and fixed with 4% paraformaldehyde at room temperature for 10 min. After discarding the paraformaldehyde, the DPBS was rinsed. The AP dye solution was added into a 1.5 mL centrifuge tube, mixed evenly, and left in the incubator for 2 min. Then, 1.10 mL of ultra-pure water was added, and 25 μL naphthol-AS-BI alkaline solution was added. The cells in the feeder layer were added with AP dye solution, kept away from light overnight, dye solution was discarded, and replaced with DPBS culture (Table 6).

#### 2.2.5. Karyotype Identification

When iPSC converged to 70~80%, it was transferred to the six-well cell culture plate. Culture medium containing 0.2 μg·mL^−1^ colchicine was continued at 37 °C and 5% CO_2_ for 6~8 h, then the cells were digested and separated with trypsin, then treated with 0.075 mol·L^−1^ KCl hypotonic solution at 37 °C for 25 min, and then fixed with fixed solution for 8 min. We centrifuged at 800 r·min^−1^ for 10 min and repeated fixation 3 times. The pre-cooled slide was tilted at 45 degrees and cell suspension was added, then the slide was brushed on an alcohol lamp, dried at room temperature, dyed with Giemsa solution, and sealed with a tablet. Finally, the number and morphological changes in chromosomes were observed under a 100× oil mirror.

#### 2.2.6. Immunohistochemical Detection

The 6th generation siPSC was used as the experimental sample, mouse-derived antibody was used as the specific antibody of related protein, and the experimental groups of OCT4, SOX2, and NANOG were set up, respectively, and the anti-mouse staining of REX1 was used as the negative control to verify whether the experiment had significant effects.

The cell line to be detected (P6) was passed into a 4-well plate treated with 0.1% gelatin, and when the cells converged to 80–90%, after DPBS cleaning, 4% paraformaldehyde was added and treated at room temperature for 20 min; after DPBS cleaning, 500 mL of IF buffer was added and treated at room temperature for 30 min.

The IF buffer was sucked out, the primary antibody was added, the secondary antibody was added in a wet box at 4 °C overnight, and the secondary antibody was incubated at room temperature away from light for 1 h, and then DAPI was added for 15 min (the secondary antibody was diluted with the IF buffer at a dilution ratio of 1:500 for both cases).

The anti-fluorescence quenching agent Vectashield was added to the sealing plate and photographed with an Olympus laser confocal microscope (Table 7 and Table 8).

### 2.3. Induced iPSC Transcriptome Sequencing of Black Bone Sheep

After the cells grew well, the cells were cracked and placed in a cryogenic storage tube. After the liquid nitrogen was quickly frozen, the dry ice was transported to the biological company for sequencing. Transcriptome sequencing results were used to compare the expression of differential genes and the pathway enrichment of related genes in SFs and iPSCs of black bone sheep.

### 2.4. Analysis of Oxidative Phosphorylation and Cell Cycle Marker Genes

Black-boned sheep SFs were used as the control group, and black-boned sheep iPSCs successfully induced through in vitro reprogramming technology were used as the experimental group. The expression characteristics of key factors involved in oxidative phosphorylation, apoptosis, and proliferation regulation were evaluated using the RT-PCR system.

This experiment employed a grouped control method for gene expression analysis. First, total RNA was extracted from both the experimental and control group samples using TRIzol reagent, and the RNA concentration and purity were measured using a UV spectrophotometer. Subsequently, the RNA was reverse transcribed into cDNA.

The experiment selected six marker genes for quantitative analysis: ATP synthase subunit beta (ATP5B), succinate dehydrogenase complex iron–sulfur subunit (SDHB), pro-apoptotic protein BAX, anti-apoptotic protein Bcl-2, cyclin-dependent kinase 1 (CDK1), and Cyclin D1 (Cyclin D1). Using cDNA from the experimental and control groups as templates, real-time fluorescent quantitative PCR was performed using the SYBR Green method. Each sample was set up with three biological replicates and three technical replicates. The internal reference gene GAPDH was used for normalization. The primer sequences are as follows (Table 9).

### 2.5. Detection of Oocyte Nuclear Transfer and Cleavage Rate

SFs and iPSC were selected as the donor cells and were used as the control group and the experimental group, respectively. Oocytes with a full, round shape, uniform color, clear cytoplasm, and uniform granular cell diffusion were extracted from fresh bovine ovaries by suction method using a 20 mL syringe and cultured in a 4-well plate at 38.5 °C in a 5% CO_2_ incubator for 22 h after maturation. The mature oocytes after 22 h of mature culture were treated with hyaluronidase for 5 min, and the cumulus cells were removed and the mature oocytes with the second polar body were selected. Then, the mature oocytes polar body and surrounding cytoplasm were sucked out by a micro-operator, and the nuclei of the donor cells were injected into the enucleated oocytes, and the two cells were fused to form a reconstructed embryo by electric fusion. After that, the reconstructed embryos were activated by simulating the physiological signals after fertilization in vivo (the reconstructed embryos were activated in ionomycin for 5 min and then put into 6DAMP for 3 h), and the reconstructed embryos were placed in the development solution 3 h later to promote their development.

Finally, the activated reconstructed embryos were cultured in a cell incubator at 38.5 °C and 5% CO_2_ for 7 consecutive days, and the cleavage rates of the control group and the experimental group were observed and recorded to evaluate the experimental effect.

### 2.6. Detection and Analysis of Expression Levels of Marker Genes in Three Layers of Early Embryo

Egg cells of SFs and iPSC of Ukrainian sheep were placed in sof medium for further culture. After 6 days, embryonic samples were collected and RNA was extracted, and gene expression levels of specific markers of the triderm were detected by RT-qPCR (Table 10), so as to explore the potential of iPSCs of Ukrainian sheep to differentiate into the triderm.

## 3. Statistical Analysis

All data 0020 and graphs were statistically analyzed using GraphPad Prism 9.3.1 Software (GraphPad Software Insightful Science, Boston, MA, USA) and SPSS 23.0 software (SPSS Inc., Chicago, IL, USA). Three independent biological replicates were designed (N = 3), and the results were expressed as percentage (mean + standard deviation). *p* < 0.05 was considered statistically significant (*** *p* < 0.001, the difference is very significant; ** *p* < 0.01, the difference is very significant; and *, *p* < 0.05, the difference is significant).

## 4. Results

### 4.1. iPSC Preparation and Identification Results of Induced Black Bone Sheep

#### 4.1.1. Analysis of Electrotransfection Efficiency

**Figure 1:**
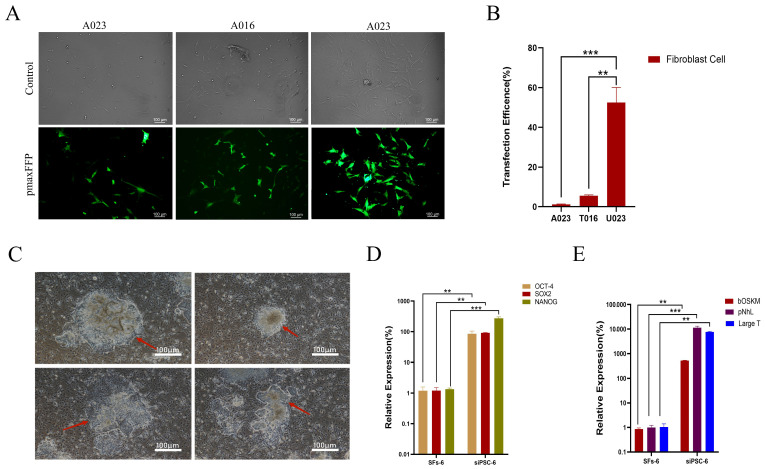
Preparation of iPSC and pluripotent gene detection of black bone sheep. (**A**) F5 female black bone sheep skin fibroblasts were compared by different electrotransfer methods (control: bright field image of transfected cells under different transfection procedures and pmaxEGFP: green fluorescence image of transfected cells under different transfection procedures, 100 μm). (**B**) Transmission efficiency of different transfection procedures (***, *p* < 0.001; **, *p* < 0.01); (**C**) clonal morphology of iPSCs in black bone sheep (red arrow indicates clonal generation edge, 100 μm); (**D**) endogenous SIPSC-29 multipotent gene detection (***, *p* < 0.001; **, *p* < 0.01); and (**E**) exogenous siPSC multipotent gene detection (***, *p* < 0.001; **, *p* < 0.01).

The transfection efficiency of U023 for SFs in black bone sheep skin was significantly higher than that of T016 (*p* < 0.01). The transfection efficiency of U023 for SFs in black-bone sheep skin was much higher than that of A023, and the difference was very significant (*p* < 0.001) (Figure 1A,B).

#### 4.1.2. Cell Morphology Analysis

After 96 h of electric transfer, the morphological changes in the cells were observed, mainly in the cells with slightly bright edges. With the extension of the culture time, the clones of the cells were raised or flattened gradually. The edges of the clones were clear and round or dispersed, showing a high nucleocytoplasmic ratio. The clones of the iPSCs of black bone sheep were raised or flattened, with clear, round, and colony-like growth at the position indicated by the arrow and distinct boundaries with the surrounding feeder cells, showing the typical characteristics of stem cells with a small cytoplasm and large nucleus (Figure 1C).

#### 4.1.3. Pluripotent Gene Detection

Three cell lines with a high endogenous gene expression were selected from 43 cell lines and named siPSC. The NANOG expression level of the endogenous siPSC was significantly higher than that of the dry wild-type NANOG, and the difference was very significant (*p* < 0.001). The SOX2 expression of the endogenous siPSC was significantly higher than that of wild-type SOX2 (*p* < 0.01). The OCT4 expression level of the endogenous siPSC was significantly higher than that of the wild-type SOX2 (*p* < 0.01) (Figure 1D).

The expression of exogenous pNhL in the siPSC cell line was significantly higher than that of the wild-type pNhL (*p* < 0.001). The expression of exogenous LargeT in the siPSC cell line was significantly higher than that of the wild-type LargeT (*p* < 0.01). The expression of exogenous bOSKM in the siPSC cell line was significantly higher than that of the wild-type bOSKM (*p* < 0.01) (Figure 1E).

#### 4.1.4. Analysis of Biological Characteristics

**Figure 2:**
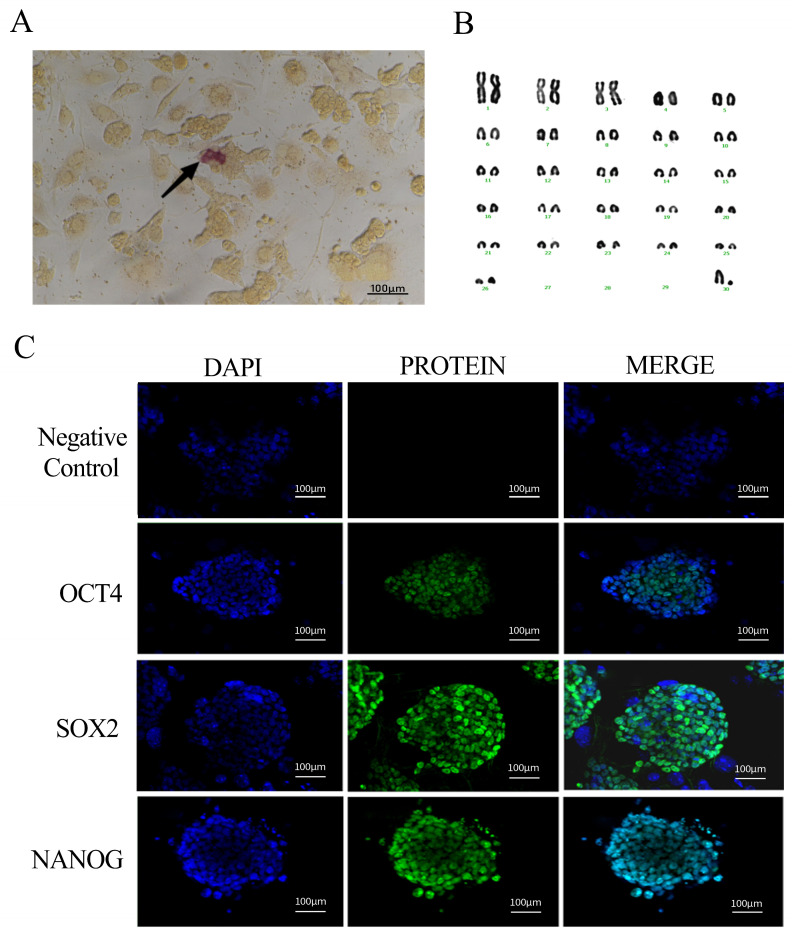
Identification of iPSC biological characteristics of black bone sheep (**A**): AP staining (100 µm); (**B**): karyotype and arrangement of iPSCs in female black bone sheep; and (**C**): siPSC pluripotent protein factor expression (nuclear: blue, protein factor: green, and fusion: blue + green) (scale: 100 µm).

The results of the iPSC alkaline phosphatase staining of the black bone sheep were weakly positive, and the precipitation formed by some cells after staining was light-purplish red, which proved that the stem cells were active. Fibroblasts were distinguished from reprogrammed cell clusters by this property, and active black bone sheep IPscs were screened out (Figure 2A).

With the increase in passage times, iPSCs are prone to karyotype abnormality. Therefore, we conducted a karyotype analysis of the six generations of sheep iPSCs, took photos at the genetic workstation of the microscope, sequentially sorted and counted the relevant parameters of chromosome size and morphology, and found that 90% of the cells showed a normal karyotype, that is, 2n = 54 (Figure 2B).

The expression characteristics of pluripotent genes in siPSCs of black bone sheep were detected by immunofluorescence labeling. The nuclei were stained with DAPI and showed a typical blue nuclear fluorescence under the excitation light, clearly showing the cytoplasmic boundary of the cells. Polyclonal antibodies against OCT4, SOX2, and NANOG were labeled with green fluorescence and showed a characteristic green fluorescence signal under the excitation light. All three pluripotent proteins showed typical nuclear localization features, which completely overlapped with DAPI-labeled nuclear regions. The experimental results showed that there were significant expressions of the pluripotent endogenous genes OCT4, SOX2, and NANOG in the siPSC of black bone sheep (Figure 2C).

### 4.2. Analysis of iPSC Transcriptome Results of Induced Black Bone Sheep

Transcriptome sequencing was performed on SFs and iPSCs of black bone sheep, and the differential expression of the iPSCs was induced by a transcriptome data comparison.

**Figure 3:**
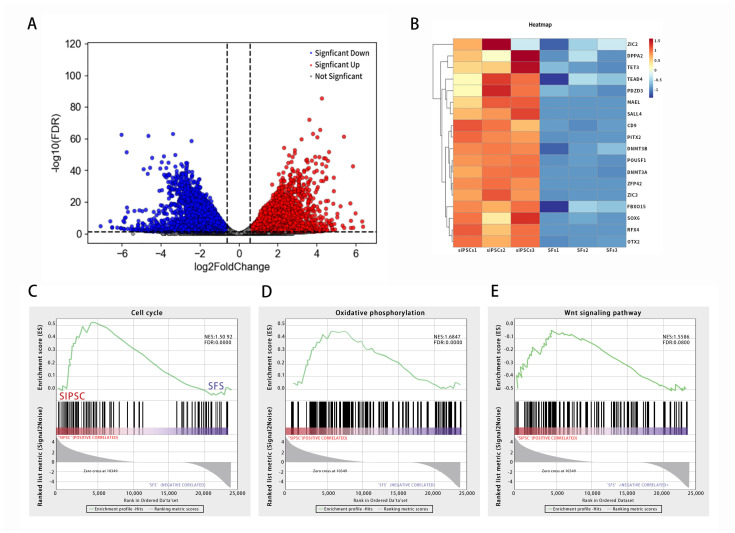
iPSC transcriptome sequencing analysis of black bone sheep. (**A**): induction of differentially expressed gene volcano in iPSC cells of black bone sheep. Red represents genes that are significantly different and up-regulated; blue represents genes that are significantly different and down-regulated; and gray represents genes that are not significantly different. (**B**): heat map shows the expression of marker genes. Red trend is up-regulated expression and blue trend is down-regulated expression. (**C**–**E**): gene set enrichment analysis (GSEA); the green line shows the enrichment profile. NES: normalized enrichment score and FDR: false discovery rate.

The volcanic map showed that the iPSCs of induced black bone sheep were significantly different from those of the control group. Among them, the expression of 256 genes was significantly up-regulated, 242 genes were significantly down-regulated, and 78 genes had no significant differences (Figure 3A). The heat map showed that the expression of Navie genes, such as TEAD4, MAEL, CD9, SALL4, and FBXO15, was up-regulated. The expression of primed multipotent marker genes, such as ZIC2, DPPA2, TET3, OTX2, and SOX6, was up-regulated in SiPSCs (Figure 3B). The signal pathway map showed that, compared with SFs, the SiPSC was significantly enriched in the oxidative phosphorylation and cell proliferation signaling pathways, and differential genes were mainly enriched in the cell cycle, oxidative phosphorylation, and Wnt signaling pathways (Figure 3C–E).

### 4.3. Oxidative Phosphorylation and Validation of Cell Cycle Marker Genes

**Figure 4:**
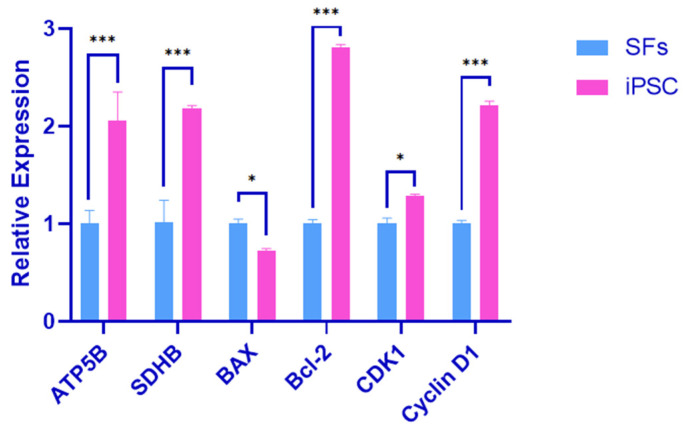
Oxidative phosphorylation and cell cycle marker gene validation. Error bars indicate three independent biological replicates (mean ± standard deviation). *** *p* < 0.001, difference is very significant; *, *p* < 0.05, difference is significant.

SFs and iPSCs were used as the control group and the experimental group. RNA was extracted from the two groups for RT-qPCR detection. The test results showed that the expression of the oxidative phosphorylation-related genes ATP5B and SDHB in the experimental group was up-regulated, and the differences were extremely significant (*p* < 0.01). The expression of the pro-apoptotic gene BAX was down-regulated, and the difference was significant (*p* < 0.05). The expression of the anti-apoptosis gene Bcl-2 was up-regulated, and the difference was significant (*p* < 0.01). The cell cycle-related gene CDK1 was up-regulated, and the difference was significant (*p* < 0.05). The Cyclin D1 expression was up-regulated and significantly different (*p* < 0.01) (Figure 4).

The results showed that the iPSC proliferation ability of black bone sheep was stronger, and it was related to the oxidative phosphorylation and cell growth-related signaling pathways MAPK, Wnt, and PI3K-AKT.

### 4.4. Cleavage Rate Detection

After the oocytes matured for 18–22 h, somatic cell nuclear transfer experiments were carried out on the SFs and iPSCs of black bone sheep, and the reconstructed embryos were developed and cultured after the experiments were completed. The cells in each group began to cleave on the second day, and the blastomes in the embryos exhibited even fission, the zona pellucida was smooth, and the development was good. The blastocyst stage of the reconstructed embryo was reached at 5–7 days. The cells were arranged closely and evenly in morphology, the blastocyst cavity was moderate in size, the boundary was clear, and its regularity could be intuitively felt in appearance, and it was ready for incubation at any time.

The results showed that 350 SFs underwent the somatic cell nuclear transfer experiment (N = 3), the cleavage rate was 83 (290 ± 3.12), and the blastocyst rate was 49 (172 ± 2.40). The cleavage rate and blastocyst rate of the 350 IPscs were 85 (297 ± 2.12) and 52 (182 ± 2.11) after the somatic cell nuclear transfer experiment (N = 3).

The remodeled iPSC was significantly higher than the SFs in the indicators of the embryo development cleavage rate and blastocyst rate (*p* < 0.01), which effectively indicated that as donor cells, iPSCs had a stronger developmental potential, which would directly affect the later embryonic development (Table 11).

### 4.5. Analysis of Early Embryo Triploblastic Marker Genes

SFs and iPSCs were used as the control group and experimental group. RNA was extracted from the two groups and the pluripotent expression of the three-layer marker genes in the iPSCs of Black Mountain sheep was analyzed by RT-qPCR.

**Figure 5:**
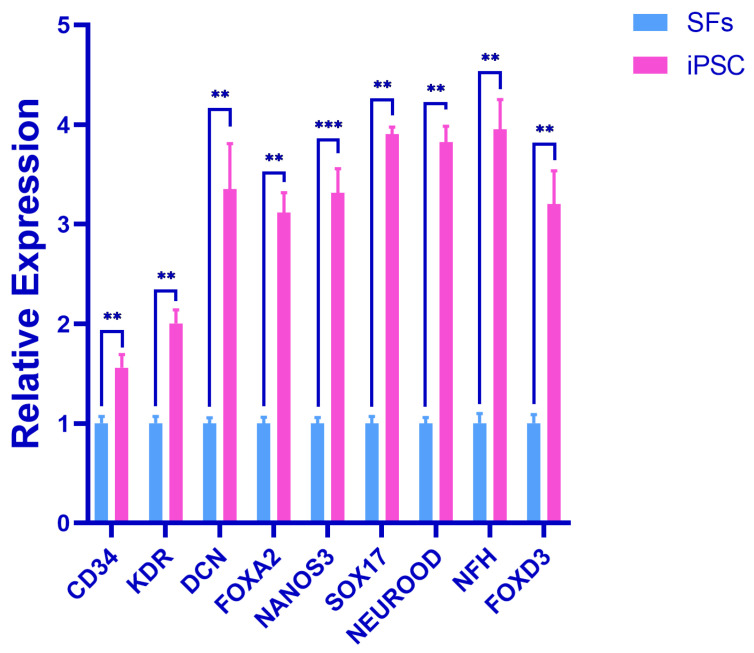
Detection of triploblastic marker genes in early embryo. Error bars indicate three independent biological replicates (mean ± standard deviation). *** *p* < 0.001, difference is very significant; ** *p* < 0.01, difference is very significant.

The results showed that, compared with the SFs in the control group, the expression levels of the endodermal marker genes (DCN, NANOS3, FOXA2, FOXD3, and SOX17), mesodermal marker genes (KDR and CD34), and ectodermal marker genes (NEUROD and NFH) were significantly increased in the experimental group. The expression difference in NANOS3 was very significant (*p* < 0.001), and the expression difference in other genes was very significant (*p* < 0.01). This suggests that the iPSC has the potential to differentiate into three germ layers (Figure 5).

## 5. Discussion

Pluripotent stem cells possess an infinite proliferative capacity and the potential to differentiate into cells of all three germ layers. Over the past few decades, studies have indicated significant similarities between teratoma cells and embryonic cells during mouse development. Stem cell lines derived from mice teratoma have provided valuable biochemistry, immunology, and genetic insights into early mammalian development [17]. Gurdon pioneered somatic cell nuclear transfer in human history by successfully transferring a frog’s somatic cell nucleus into an oocyte [18]. Following the birth of Dolly, the cloned sheep, in 1997, somatic cell nuclear transfer was successfully applied to various species, including cattle, mice, pigs, and sheep [19]. In 2006, Yanmanaka et al. introduced a groundbreaking technique that transports the four transcription factors, including OCT4, SOX2, KLF4, and c-MYC, into SFs to induce the formation of pluripotent stem cells [20]. It bypassed the ethics controversies associated with embryonic stem cells and opened new avenues for livestock stem cell research.

The induction of exogenous OSKM transcription factors into somatic cells leads to a constant expression of both exogenous OCT4 and SOX2, as well as endogenous OCT4, SOX2, and NANOG. These factors also activate core pluripotency transcriptional networks. These factors play indispensable roles in maintaining pluripotency and promoting early embryonic development by activating pluripotency genes while repressing genes related to differentiation. Although KLF4 and cMyc are not strictly essential for reprogramming, they will reduce the effect of the process. KLF4 is pivotal in cellular development, proliferation, and apoptosis [21]. It can restore the epidermis-derived stem cells to an embryonic stem cell state and works synergistically with the OCT4–SOX2 complex to promote iPSC generation. cMyc, an oncogene, regulates the cell cycle and drives cell division [22]. It facilitates epigenetics in somatic cells, accelerating the reprogramming process. While OSKM transcription factors are typically sufficient for reprogramming, this study applied Lin-28 and SV 40 Large T to enhance stability and reprogramming efficiency.

The preliminary transcriptome analysis using the reference genome revealed 73 significantly enriched pathways in the KEGG database, many of which are closely related to stem cell function, including the MAPK, PI3K-AKT, and Wnt signaling pathways. The MAPK pathway is a fundamental signal transduction system that mediates cellular responses to external stimuli and is tightly linked to oxidative phosphorylation. The ATP5B gene encodes the β-subunit of ATP synthase, a key enzyme in oxidative phosphorylation that catalyzes ATP production. The β-subunit participates in the catalysis of generating ATP. The SDHB gene is a composition of succinate dehydrogenase (complex II), which is involved in the tricarboxylic acid cycle and respiratory chain electron transport, thus contributing to oxidative phosphorylation [23]. The PI3K-AKT and Wnt signaling pathways collaboratively regulate cellular proliferation and programmed cell death [24]. Regarding apoptosis, BAX, a proapoptotic gene, encodes the BAX protein that forms mitochondrial membrane channels, facilitating the release of proapoptotic factors, such as cytochrome, to the cytoplasm from mitochondria and activating downstream apoptosis signaling pathways. Conversely, BCL-2, an antiapoptotic gene, encodes the BCL-2 protein that can inhibit the release of cytochrome c, thus preventing apoptosis. The balance of the expression level and interactions of BAX and BCL-2 is of significant importance for apoptosis regulation. During the process of cycle regulation, CDK1, which is located in the PI3K-AKT signaling pathway, is a core component in orchestrating ordered cell division. CDK1, along with Cyclin D1, is a fundamental factor in cellular cycle regulation [25]. This study suggests that the gene expression of ATP5B, SDHB, BCL-2, CDK1, and Cyclin D1 up-regulated significantly while the BAX gene down-regulated remarkably. These gene expression changes enhance oxidative phosphorylation, accelerate cell cycle progression, promote proliferation, and inhibit apoptosis, collectively improving cell growth and viability.

iPSCs can express core pluripotency genes, including OCT4, SOX2, and NANOG. Nuclear transfer can accelerate embryonic genome activation and decrease dependence on maternal factors, thereby improving the synchronicity of the cleavage and blastocyst formation. Due to the undifferentiated state of iPSCs, their cell cycle predominantly resides in the G1 stage, which is more likely to synchronize with enucleated oocytes in the M stage. This synchronization reduces the risk of DNA damage after nuclear transfer and increases the normal chromosome segregation rate. Additionally, iPSCs may better adapt to the energy demands during the early embryo development stage through metabolic reprogramming, while SFs, as terminally differentiated cells, may fail to respond rapidly to fluctuating energy requirements. Consequently, iPSCs as donor cells exhibited a very significant increase in the cleavage and blastulation rate compared with SFs, showing a superior early embryonic developmental potential. These findings indicate that iPSCs can serve as a more effective donor cell source for somatic cell nuclear transfer, offering a new strategy for the cloning or preservation of genetic resources of black bone sheep.

The iPSC is capable of differentiating into three germ layers (ectoderm, mesoderm, and endoderm) in vitro, similarly to embryonic stem cells. Under specific conditions and with the addition of proper growth factors, the iPSC can be induced to differentiate into neural precursor cells that express neuroectodermal-specific markers, such as NEUROD and NFH [26]. These neural precursor cells can further differentiate into various neural cells, including neurons, astrocytes, and oligodendroglia. Specific growth factors and signaling factors can be added, such as bone morphogenetic protein (BMP) and fibroblast growth factors (FGFs) [27], to induce the differentiation of mesoderm cells. The iPSC first differentiates into mesoderm precursor cells, which express specific markers, such as KDR and CD34, which can subsequently develop into cardiomyocytes, vascular endothelial cells, chondrocytes, osteoblasts, and others. Endodermal differentiation requires Activin A, Wnt3a, and other specific growth factors and signaling factors [28]. iPSC-derived endoderm precursor cells express markers like DCN, NANOS3, FOXA2, FOXD3, and SOX17 and can additionally differentiate into hepatocytes, pancreatic cells, and pulmonary cells.

In the field of life science, the iPSCs of black bone sheep have a broad potential application value. From the perspective of agricultural improvement, iPSCs of black bone sheep provide a unique research model for further exploring the mechanism of cell reprogramming and the mechanism of pluripotency maintenance. Studies have shown that the study of iPSCs in sheep is helpful to understand the conserved and specific mechanisms in the process of cell reprogramming between different species [29]. The iPSC technology can promote the cryopreservation of somatic cells and reprogram them into pluripotent stem cells, establish a germplasm bank, and reveal new molecular mechanisms of cell reprogramming and pluripotent maintenance under a specific genetic background. It is of great significance to improve the theoretical basis of cell-related technology in agricultural improvement. In terms of clinical application prospects, due to certain similarities between the iPSCs of black bone sheep and humans in terms of physiological structure and metabolism, ipscs of black bone sheep can be used as a large animal model for preclinical research, and all kinds of cells can be differentiated for transplantation experiments, so as to evaluate the safety and effectiveness of cell therapy and provide an important reference for future human cell therapy [30]. In addition, the iPSCs of black bone sheep provide a powerful tool for studying gene function and disease pathogenesis. By manipulating the iPSCs of black bone sheep through gene editing technology, cell models carrying specific gene mutations are constructed, which can simulate the occurrence and development of human genetic diseases and lead to the further study of the molecular mechanism of diseases [31].

Overall, the pluripotency of black bone sheep iPSCs involves intricate signal regulatory networks. The expression of pluripotency-associated genes is related to MAPK, P13K-AKT, and the Wnt signaling pathway. They can also effectively promote the proliferation of cells. The observed high early embryo cleavage rates and the expression of germ-layer-specific markers confirm the potential of black bone sheep iPSCs to contribute to embryo development following nuclear transfer.

In this study, the piggyBac+TET-on transposon subsystem was utilized to induce fibroblast reprogramming in black bone sheep by transmitting seven exogenous reprogramming factors (OCT4, SOX2, KLF4, cMyc, NANOG, Lin-28, and SV40 Large T). The resulting induced pluripotent stem cells (iPSCs) exhibit the expression of pluripotent genes, participate in the three-line differentiation in vitro, and promote the early embryonic development of sheep. Transcriptomic analysis has revealed new insights into reprogrammed iPSCs, laying the foundation for the construction of sheep pluripotent stem cells and their role in early embryonic development. However, in practical applications, the developmental potential of reconstructed embryos after transplantation into the uterus has not yet been evaluated. The applicability of the piggyBac+TET-on system in other sheep breeds or large animals needs to be further verified. Future research will further explore the power of research and development to provide theoretical support for the breeding of large animals.

## 6. Conclusions

This study successfully reprogrammed black-boned sheep fibroblasts into induced pluripotent stem cells (iPSCs) by introducing seven exogenous reprogramming factors (OCT4, SOX2, KLF4, cMyc, NANOG, Lin-28, and SV40 Large T) using the piggyBac+TET-on transposon system. Transcriptome analysis revealed that iPSCs were significantly enriched in oxidative phosphorylation and cell proliferation pathways, with an up-regulated expression of related genes such as ATP5B, SDHB, Bcl-2, CDK1, and Cyclin D1, while the pro-apoptotic gene BAX was significantly down-regulated, indicating that iPSCs possess strong proliferative and anti-apoptotic capabilities. Additionally, somatic cell nuclear transfer experiments demonstrated that the cleavage and blastocyst rates of iPSCs were significantly higher than those of fibroblasts, suggesting that iPSCs have greater developmental potential in early embryonic development. The further detection of trilineage marker genes confirmed that iPSCs have the ability to differentiate into all three germ layers, with the expression levels of endodermal, mesodermal, and ectodermal marker genes being significantly higher than those in the control group. In summary, this study successfully constructed pluripotent black-boned sheep iPSCs, elucidated their role in early embryonic development, and provided a theoretical foundation for the construction of sheep pluripotent stem cells and their applications in animal production and genetic resource preservation.

## Figures and Tables

**Table 1:** Preparation of fibroblast culture medium.

Component Name	Volume (mL)
DMEM/F12	445 mL
100 × Penicillin–streptomycin	5 mL
Extra fetal bovine serum	50 mL

**Table 2:** Preparation of M10 medium (STO cell culture).

Component Name	Volume (mL)
Knockout DMEM	500
100 × Penicillin–streptomycin	5
100 × GlutaMAX Supplement	5
100 × MEM Non-essential Amino Acids Solution	5
Extra fetal bovine serum	57

**Table 3:** Preparation of M15 medium (induced stem cell differentiation).

Component Name	Volume (mL)
DMEM/F12	500
Extra fetal bovine serum	93.75
Glutamine (200 mM)	6.25
100× Non-essential amino acid	6.25
100× Penicillin–streptomycin	6.25
LIF	1000 U/mL

**Table 4:** Endogenous pluripotent gene primers.

Primer Name	Primer Sequence (5′-3′)	Amplification Length
GAPDH	F: ACGGGAAGCTCACTGGCATGG	227 bp
	R: GCCAGCCCCAGCATCGAAG	
OCT-4	F: TACACTGTACTCTTCGGTCCCATT	277 bp
	R: AGCATCATTGAACTTCACCTTCCC	
SOX2	F: TACGGTAGGAGCTTTGCAGAAAGT	246 bp
	R: TGCACGTTTGCAACTGTCCTAAAT	
NANOG	F: CAAGTATTTCAGTTCCCAGCAGCA	222 bp
	R: TCCCTCAAACTGACACAGAAGGTA	

**Table 5:** Primers for exogenous gene detection.

Primer Name	Primer Sequence (5′-3′)	Amplification Length
GAPDH	F: ACGGGAAGCTCACTGGCATGG	227 bp
	R: GCCAGCCCCAGCATCGAAG	
bOSKM	F: CCGCATGTTAGCAGACTTCC	232 bp
	R: GAGGATTTTGAGGCTGCTGG	
pNhL	F: TATGTGAACCGGTGCTAGCC	180 bp
	R: TTTGGCGAGAGGGGAAAGAC	
Large T	F: CCCTTGGACAGGCTGAACTTTGAG	86 bp
	R: TCCCCTCCAGTGCCCTTTACATC	

**Table 6:** AP dye formula.

Component Name	Volume (μL)
Sodium nitrite	25
FRV alkaline solution	25

**Table 7:** Dilution ratio of primary antibody.

Antibody Name	Diluent	Dilution Ratio
OCT4 anti-mouse	IF Buffer fluid	1:200
SOX2 anti-mouse	IF Buffer fluid	1:500
NANOG anti-mouse	IF Buffer fluid	1:50
REX1 anti-mouse	IF Buffer fluid	1:100

**Table 8:** Dilution ratio of secondary antibody.

Antibody Name	Diluent	Dilution Ratio
anti-mouse	IF Buffer fluid	1:500

**Table 9:** Primers for oxidative phosphorylation and cell cycle specific marker gene detection.

Primer Name	Primer Sequence (5′-3′)
ATP5B	F CCTGAAAGATGCCACCTCCAAG
	R GCCACCGTCAGCCCAGTC
SDHB	F GCAGTCCATAGAAGAGCGTGAG
	R TGTCTCCGTTCCACCAGTAGC
BAX	F CAGGATGCGTCCACCAAGAAG
	R GTCCACGGCGGCAATCATC
Bcl-2	F CGAGTGGGATGCGGGAGATG
	R CGGGATGCGGCTGGATGG
CDK1	F TTCAGGATGTGCTTATGCAGGATTC
	R CCATGTACTGACCAGGAGGGATAG
Cyclin D1	F AGAGGCGGAGGAGAACAAACAG
	R GGAGGGCGGATTGGAAATGAAC

**Table 10:** Primers for the detection of three-layer specific markers.

Primer Name	Primer Sequence (5′-3′)
DCN	F AACAATATCTCTGCAATCGGCTC
	R AGTTTCCAAGCTGAACAGCAGC
NEUROD	F TCTTTCAAACACGAACCGTCCG
	R CGTGAAAGATGGCATTGAGCTG
NANOS3	F ACCTTCAGTCGCCCACCTAGC
	R AGCATTCGCCAGCACCTTGATC
SOX17	F TACGCCAGTGACGAGCAGAGC
	R CGCCTCGCCCTTCATCTTCATG
CD34	F AGTGCCTGCTGCTGGTCCTG
	R CTGGTGGCTGCTGACATCTTCTTC
KDR	F GGGTCCTGAAATCACGTTGCA
	R AGTCTTCCTGTCCTGAGCAAAGC
NFH	F GAGTGAGGCTGGACCGACTG
	R AGCTCAGAGCGCTGCCTCTC
FOXA2	F AGCACCACTACGCCTTCAAC
	R GGAGTACACGCCCTGGTAGTAG
FOXD3	F CTGAGCGGCATCTGCGAGTTC
	R GCGAGAGGTTGTGGCGGATG

**Table 11:** Number of early embryo cleavage.

Sample	Number of Somatic Cell Nuclear Transfer Cells (Repeats)	Development Situation (%)					
		2cell	4cell	8cell	16cell	Morula	Blastocyst
Control	350 (3)	83 (290 ± 3.12) ^b^	81 (285 ± 3.02)	78 (273 ± 0.22)	60 (210 ± 1.11)	52 (182 ± 1.22)	49 (172 ± 2.40) ^a^
Treated	350 (3)	85 (297 ± 2.12) ^b^	85 (297 ± 2.12)	80 (280 ± 0.43)	73 (256 ± 0.34) ^a^	66 (231 ± 2.42)	52 (182 ± 2.11)

Note: ^a^ indicates significance (n < 0.01) and ^b^ indicates significance (*p* < 0.05). Three independent biological replicate statistics (n = 3, percentage (mean + standard deviation)).

## Data Availability

Data are contained within the article.
